# Association Between Cardiometabolic Index and Mortality Among Patients with Atherosclerotic Cardiovascular Disease: Evidence from NHANES 1999–2018

**DOI:** 10.3390/medicina61061064

**Published:** 2025-06-10

**Authors:** Duo Yang, Wei Li, Wei Luo, Yunxiao Yang, Jiayi Yi, Chen Li, Hai Gao, Xuedong Zhao

**Affiliations:** Department of Cardiology, Beijing Anzhen Hospital, Capital Medical University, Beijing 100029, China

**Keywords:** atherosclerotic cardiovascular disease, cardiometabolic index, mortality, National Health and Nutrition Examination Survey

## Abstract

*Background and Objectives:* Atherosclerotic cardiovascular disease (ASCVD) remains a leading cause of global morbidity and mortality. The cardiometabolic index (CMI) has been shown to be associated with metabolic disorders and mortality in general populations, but its role in ASCVD-specific mortality risk remains unexplored. *Materials and Methods:* This cohort study was based on the National Health and Nutrition Examination Survey (NHANES). Weighted Cox proportional hazards models were fitted to estimate the associations between CMI and mortality. Restricted cubic splines were used to explore nonlinear relationships. Subgroup analyses were used to investigate potential differences among specific ASCVD patients. *Results:* A total of 2157 patients with ASCVD were included. Over a median 83-month follow-up, 887 all-cause and 300 cardiovascular deaths occurred. Each unit increase in CMI was associated with an 11.3% increased risk of all-cause mortality (HR = 1.113, 95% CI: 1.112–1.115) and a 6.4% increased risk of cardiovascular mortality (HR = 1.064, 95% CI: 1.062–1.065). There was a nonlinear J-shaped relationship between CMI and all-cause mortality, while the risk of cardiovascular mortality increased linearly with increasing CMI. *Conclusions:* These findings underscore the importance of monitoring and managing CMI in patients with ASCVD in clinical practice and suggest that optimizing CMI levels may help reduce the risk of death and improve the long-term prognosis of patients.

## 1. Introduction

Atherosclerotic cardiovascular disease (ASCVD) is the leading cause of hospitalization and cardiovascular death. Despite advances in therapeutic strategies, the burden of ASCVD is further exacerbated by an aging population and unhealthy lifestyle trends. Overall, global CVD deaths have increased from 12.4 million in 1990 to 19.8 million in 2022 [1]. Therefore, early detection and assessment of mortality risk in patients with ASCVD is essential for the development of effective prevention and treatment strategies.

The cardiometabolic index (CMI) is a composite index of body fat distribution and serum lipid levels, which was originally developed as a metabolic marker to predict the risk of diabetes [2]. The CMI has now been shown to be associated with the risk of hypertension, diabetes, metabolic syndrome (MetS), and stroke [3,4,5,6,7], as well as all-cause and cardiovascular mortality in the elderly population [8]. The CMI integrates several factors closely related to metabolism, such as waist-to-height ratio (WHtR), triglycerides (TG), and high-density lipoprotein cholesterol (HDL-C), which can more comprehensively reflect the metabolic status and cardiovascular risk of patients, and helps to provide a new dimension for the assessment of mortality risk in patients with ASCVD from a metabolic perspective [9]. However, to date, no study has reported the association between CMI and mortality risk in patients with ASCVD.

Using data from the National Health and Nutrition Examination Survey (NHANES), this study conducted a longitudinal cohort study to examine the associations between CMI and all-cause and cardiovascular mortality in patients with ASCVD and, further, to explore the differences in these associations in subgroups of patients with ASCVD. We hypothesize that CMI may be a useful tool for identifying high-risk individuals with ASCVD, allowing physicians to more accurately stratify their patients into risk categories, thereby allowing more rational allocation of scarce health resources to high-risk patients and improving the targeting and effectiveness of medical interventions.

## 2. Materials and Methods

### 2.1. Study Design and Population

This longitudinal cohort study was based on the NHANES database, a comprehensive survey conducted by the National Center for Health Statistics (NCHS) of the Centers for Disease Control and Prevention (CDC) in the United States. A detailed description of the study design is available on the NHANES website (https://wwwn.cdc.gov/nchs/nhanes (accessed on 1 December 2024)). In brief, the NHANES used a stratified multistage random sampling design to construct a representative sample of the U.S. population and collected data through standardized in-home interviews. The NHANES survey protocol was approved by the Research Ethics Review Board of the National Center for Health Statistics, and all participants provided written informed consent.

**Figure 1:**
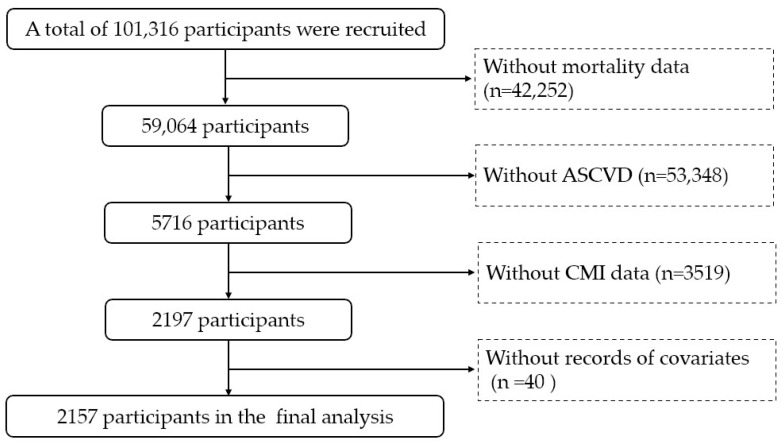
Flow chart. ASCVD: atherosclerotic cardiovascular disease. CMI: cardiometabolic index.

This study included all ASCVD patients with mortality data from 10 NHANES cycles between 1999–2000 and 2017–2018. The definition of ASCVD includes coronary heart disease (CHD), angina, heart attack, and stroke. Individuals lacking mortality data (*n* = 42,252), ASCVD diagnosis (*n* = 53,348), CMI measurements (*n* = 3519), or covariate information (*n* = 40) were excluded from the study. Finally, 2157 patients with ASCVD were included in the final analysis (Figure 1).

### 2.2. Assessment of CMI

The formula for calculating CMI was CMI = TG (mmol/L)/HDL-C (mmol/L) × waist circumference (WC, cm)/height (cm) [2]. TG, HDL-C, WC, and height data were collected through health screenings and laboratory tests. In this study, CMI was considered a continuous variable in the statistical analysis and was also divided into four groups according to the quartiles, which were <0.40 (Q1), 0.40–0.68 (Q2), 0.69–1.14 (Q3), and ≥1.15 (Q4).

### 2.3. Assessment of Mortality

The primary outcomes of this study were all-cause and cardiovascular death. Information on the date and cause of death was obtained by linkage to the National Death Index up to the end of 2019. Underlying causes of death were determined using the International Classification of Diseases, Tenth Revision (ICD-10). Cardiovascular mortality was identified as death due to heart disease (I00–I09, I11, I13, I20–I51) and cerebrovascular disease (I60–I69). The follow-up period was calculated from the date of the interview or examination to the date of death or the end of the study (31 December 2019).

### 2.4. Covariates

Covariates were collected through standardized questionnaires, health examinations, and laboratory tests, including age, gender, race, educational level, marital status, poverty income ratio (PIR), smoking status, alcohol consumption, physical activity, body mass index (BMI), total cholesterol (TC), history of hypertension, history of diabetes, and medication use.

Race was designated as Mexican American, other Hispanic, non-Hispanic White, non-Hispanic Black, and other race. Educational level was categorized as less than high school, high school, and more than high school. Marital status was classified as never married, married or living with partner, and divorced, separated, or widowed. PIR was categorized into three groups: <1.3, 1.3–3.5, and >3.5. Smoking status (never, former, and current) was defined according to the questions “Smoked at least 100 cigarettes in life?” and “Do you now smoke cigarettes?”. Drinking status (<12 drinks/year and ≥12 drinks/year) was determined by asking participants whether they consumed alcohol at least 12 times per year. Moderate-to-vigorous physical activity (MVPA) was defined as participation in vigorous or moderate physical activity within the past 30 days, or participation in vigorous or moderate work activity. BMI was calculated by dividing weight in kilograms by the square of height in meters (kg/m^2^). Information on medical history and medication history was based on patient self-reports.

### 2.5. Statistical Analysis

Weighted analyses were performed based on the complex sampling survey, in accordance with NHANES recommendations. Data were expressed as mean ± standard deviation (SD) or median (interquartile range [IQR]) for continuous variables, and frequency with percentage for categorical variables. Weighted one-way analysis of variance (ANOVA), the Kruskal–Wallis H test, and the chi-square test were used to compare the differences between groups.

Kaplan–Meier survival curves were used to estimate survival for baseline CMI quartiles, with the log-rank test used to compare different survival curves. Weighted univariate and multivariate Cox proportional hazards models were fitted to estimate the associations between CMI and all-cause and cardiovascular mortality. Model 1 was unadjusted. Model 2 was adjusted for age, gender, and race. Model 3 was further adjusted for educational level, marital status, PIR, smoking status, alcohol consumption, MVPA, BMI, TC, hypertension, diabetes, and medication use. The results of the Cox regression models are presented as hazard ratios (HRs) and 95% confidence intervals (CIs). The proportional hazards assumption for each variable was tested using Schoenfeld residuals, and no violation was identified (*p* > 0.05). A restricted cubic spline with 3 knots was used to explore the potential nonlinear association between CMI and mortality [10].

Subgroup analyses were used to investigate potential differences among specific ASCVD patients, including gender, educational level, marital status, family PIR, smoking status, alcohol consumption, MVPA, hypertension, and diabetes.

All analyses were performed using SAS (version 9.4) and R (version 3.5.1). A two-sided *p* value < 0.05 was considered statistically significant.

## 3. Results

### 3.1. Baseline Characteristics

A total of 2157 patients with ASCVD were included in this study, with a mean age of 66.4 ± 12.6 years, and 57.7% were male. Baseline characteristics of the patients are shown in Table 1. The median CMI was 0.69 (IQR 0.40–1.15), with a range of 0.04 to 6.57. Patients were categorized into four groups according to the CMI quartiles. Compared with Q1 of CMI, the higher quartiles exhibited a higher proportion of males, a greater representation of Mexican American and non-Hispanic White ethnicity, a lower proportion of higher education, a lower family PIR, a higher proportion of smokers, a higher BMI and TC, a higher proportion of diabetes, and higher rates of taking antihypertensive, antidiabetic, and cardiovascular medications.

### 3.2. Association Between CMI and Mortality

**Figure 2:**
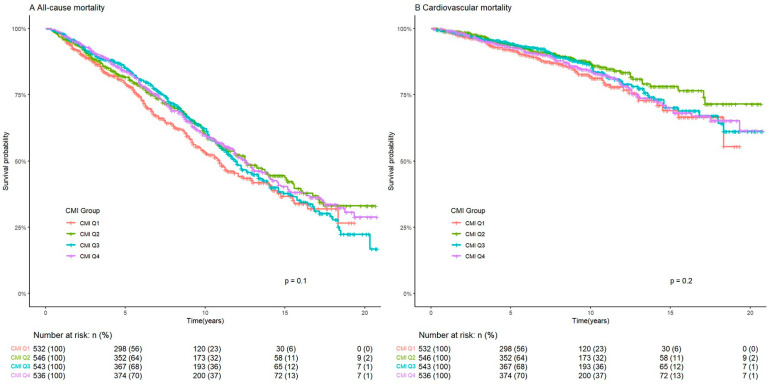
Kaplan–Meier curves of the survival rate of ASCVD patients with CMI groups. ASCVD: atherosclerotic cardiovascular disease. CMI: cardiometabolic index.

During a median follow-up period of 83 months, there were 887 all-cause deaths and 300 cardiovascular deaths. Kaplan–Meier curves for the survival rate of ASCVD patients in the CMI groups are shown in Figure 2. Patients in the CMI Q2 group had the lowest all-cause and cardiovascular mortality, but the difference in mortality between the four groups is not significant (*p* values are 0.1 and 0.2, respectively).

The results of the weighted Cox regression models are shown in Table 2. In the fully adjusted model, each unit increase in CMI was associated with an 11.3% (HR = 1.113, 95% CI: 1.112–1.115) increased risk of all-cause mortality and a 6.4% (HR = 1.064, 95% CI: 1.062–1.065) increased risk of cardiovascular mortality. The HRs for all-cause mortality were 1.426 (95% CI: 1.423–1.430), 1.332 (95% CI: 1.328–1.335), and 1.426 (95% CI: 1.422–1.430) in the Q1, Q3, and Q4 groups, respectively, compared with the CMI Q1 group. The HRs for cardiovascular mortality were 1.678 (95% CI: 1.670–1.686), 1.397 (95% CI: 1.391–1.404), and 1.826 (95% CI: 1.818–1.835) in the Q1, Q3, and Q4 groups, respectively, compared with the CMI Q1 group.

**Figure 3:**
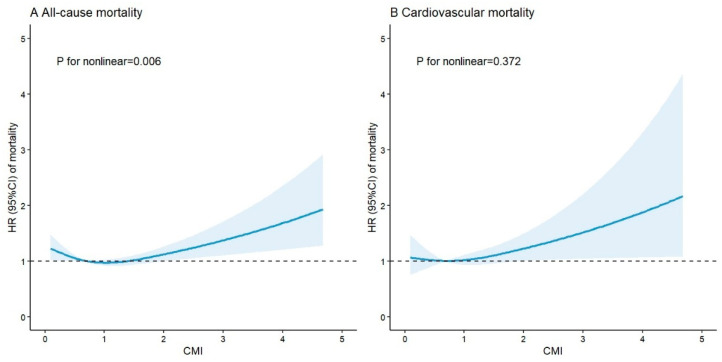
Restricted cubic splines for the association between CMI and mortality in ASCVD patients. The horizontal dotted line represents the HR of 1.0. HRs are indicated by solid blue lines and 95% CIs by blue areas. Model adjusted for age, gender, race, educational level, marital status, family PIR, smoking status, alcohol consumption, MVPA, BMI, TC, hypertension, diabetes, and medication use. CMI: cardiometabolic index; ASCVD: atherosclerotic cardiovascular disease; HR: hazard ratio; 95% CI: 95% confidence interval; PIR: poverty income ratio; MVPA: moderate-to-vigorous physical activity; BMI: body mass index; TC: total cholesterol.

As shown in Figure 3, there was a nonlinear J-shaped relationship between CMI and all-cause mortality (*p* for nonlinear was 0.006). The risk of all-cause mortality increased when the CMI was less than 0.67 or greater than 1.43. In addition, the risk of cardiovascular mortality increased with increasing CMI (*p* for nonlinear was 0.372).

### 3.3. Subgroup Analyses

The results of the subgroup analyses are shown in Table 3. For all-cause mortality, the association between CMI and mortality risk was stronger in females, former smokers, and those with a low educational level, participation in MVPA, hypertension, and diabetes. For cardiovascular mortality, CMI was more strongly associated with mortality risk in males, former smokers, and those with a low educational level, participation in MVPA, hypertension, and diabetes. All *p* values for interaction were <0.001.

## 4. Discussion

This study demonstrates a significant association between CMI and the risk of mortality in patients with ASCVD. This association exhibits a nonlinear J-shaped pattern for all-cause mortality. The increased risk of mortality associated with CMI was more pronounced in patients with ASCVD who had a low level of education, were former smokers, participated in MVPA, and had hypertension and diabetes mellitus.

Previous studies have demonstrated the significant association between CMI and several diseases. As a metabolic marker which was originally developed to predict the risk of diabetes, CMI has been reported to be associated with insulin resistance, impaired fasting glucose, and diabetes [4,11,12]. The association between CMI and MetS has also been extensively studied, for example, two studies in Caucasian adults indicated that CMI was one of the most useful indices in detecting MetS [5,6]. A study based on the Multi-Ethnic Study of Atherosclerosis (MESA) found that the greater the increase in CMI from 2000 to 2005, the higher the risk of CVD [13]. A Japanese study of 63 patients with peripheral arterial disease reported that CMI was associated with atherosclerotic progression [14]. Another study in Chinese patients with diabetes also suggested that CMI was a relevant and independent marker of atherosclerosis [15]. In addition, several studies have examined the impact of CMI on mortality risk in the general population. A recent study of 3752 individuals reported that CMI was significantly associated with both all-cause and cardiovascular mortality in American adults over 40 years of age [16]. Similarly, a study based on six NHANES cycles showed a positive association between elevated CMI and all-cause mortality among 3029 participants over the age of 65 [8]. Wang J. et al. found a positive association between CMI and all-cause and cancer mortality, and a negative association with cardiovascular mortality [17]. Although the results of the above studies are not entirely consistent due to differences in the study methodology of the populations studied, our results and those of previous studies suggest that CMI has significant value in predicting the risk of disease and death.

To our knowledge, this is the first study to investigate the association between CMI and mortality in patients with ASCVD. In this study, we found a J-shaped nonlinear association between CMI and all-cause mortality. The CMI was significantly associated with increased all-cause mortality when it was below 0.67 and above 1.43, whereas the association disappeared between 0.67 and 1.43, suggesting that both low and high levels of CMI may have distinct effects on patient outcomes. This pattern is similar to the findings in the general population, where the dose–response relationship between CMI and all-cause mortality was L-shaped [17,18]. There are several possible mechanisms that could explain this phenomenon. Patients with a low CMI at the end of the spectrum may have poor metabolic health, as evidenced by conditions such as weakness or malnutrition, which could increase mortality [19]. On the other hand, MetS, insulin resistance, and systemic inflammation—all known risk factors for acute cardiovascular disease and all-cause mortality—are often associated with a high CMI [20,21,22,23]. Additionally, our study also found that the association between CMI and CVD mortality exhibited a similar J-shaped pattern. However, the nonlinearity test was not statistically significant due to the relatively small number of cardiovascular death events. In consideration of the potential clinical implications of these findings, further research in this area is warranted.

The findings highlight the clinical importance of monitoring and managing CMI in patients with ASCVD. In clinical practice, CMI could serve as a valuable adjunct to existing risk stratification tools, it could help clinicians better identify patients at higher risk of adverse outcomes who may require more intensive monitoring or interventions. According to this J-shaped association, in order to properly identify and address related illness concerns, health assessments should focus on a moderate range of the CMI. The CMI, a composite index integrating TG, HDL-C, WC, and height, can be modulated through lifestyle interventions. Dietary modification and physical activity, for instance, have demonstrated efficacy in improving metabolic health and reducing ASCVD risk [24,25,26]. For patients with a lower CMI, the focus should be on improving general health and providing nutritional support. In contrast, for patients with a higher CMI, the focus should be on controlling the components of MetS, including blood glucose, lipid, and blood pressure levels.

Subgroup analyses revealed significant sex differences in the association of CMI with mortality. Specifically, the association of CMI with all-cause mortality was stronger in females, whereas the association with cardiovascular mortality was stronger in males. Numerous physiologic, epidemiologic, and therapeutic factors could contribute to the sex differences in this association. For example, males tend to have more traditional cardiovascular risk factors than females [27]. While smoking is a well-established risk factor for mortality [28] and may exacerbate metabolic disorders by increasing insulin resistance, inflammatory responses, and oxidative stress [29,30], our stratified analysis revealed that, for both all-cause mortality and cardiovascular mortality, the effects of CMI are stronger in former smokers. This finding may reflect survivor bias, wherein former smokers who successfully quit represent a subgroup with persistent metabolic vulnerability, potentially exacerbated by smoking-induced epigenetic modifications that perpetuate insulin resistance and amplify CMI-related risks. Although this observation underscores the complex interplay between smoking history and metabolic risk, further mechanistic studies are needed to disentangle whether smoking cessation augments susceptibility to CMI-driven pathways or reflects selective survival of high-risk individuals. Notably, we also found that the effects of CMI on mortality risk in ASCVD patients were particularly significant in individuals with hypertension or diabetes, which may be related to the synergistic effects of glucose and lipid dysregulation, chronic inflammation, and hemodynamic stress. Interestingly, the mortality risk of CMI was significantly higher in patients who regularly engaged in MVPA. The data show higher rates of smoking (63.3% vs. 61.2%) and lower rates of cardiovascular medication use (66.0% vs. 70.7%) among those who participate in MVPA, which may contribute to the higher risk of mortality.

The mechanisms underlying the association between CMI and the increased risk of death in patients with ASCVD are currently unclear. It is hypothesized that the possible mechanisms involve multiple pathophysiological interactions. First, metabolic disturbances are one of the central mechanisms. Elevated CMI and its components are usually accompanied by insulin resistance, hyperglycemia, dyslipidemia, and abdominal obesity, and these metabolic disturbances contribute to atherosclerosis [2,31,32,33,34]. Second, inflammation has been reported to be a significant mediator of the association between CMI and mortality in the elderly population, with leukocytes and neutrophils mediating 6.6% and 13.9% of the association, respectively [8]. In addition, chronic inflammation exacerbates the process of atherosclerosis, thereby increasing the risk of mortality in patients with ASCVD [35,36]. Finally, endothelial dysfunction and oxidative stress due to metabolic abnormalities are also strongly associated with adverse cardiovascular outcomes [37,38]. The combined effect of these mechanisms may explain the significant association between CMI and mortality.

The present study has several limitations. First, as an observational study, the results cannot conclusively prove causality. Second, despite adjustment for several covariates, residual and unmeasured confounding may still exist and affect the results. Third, information on CVD, hypertension, diabetes, and medication use was collected via self-report questionnaires, which may introduce recall bias. Participants might inaccurately recall or report their medical history and medication use, potentially leading to misclassification of these variables. This misclassification could affect the precision of our estimates and might either attenuate or exaggerate the observed associations between CMI and mortality outcomes. In addition, the NHANES database only collects information on whether participants had at least 12 drinks per year, and thus we are unable to further differentiate between infrequent and regular drinkers. This limitation may obscure potential dose–response relationships between alcohol consumption and mortality outcomes in patients with ASCVD. Fourth, the study did not consider the effect of dynamic changes in CMI during follow-up on the risk of mortality. Fifth, the results regarding cardiovascular mortality and the subgroup analyses should be treated with caution due to the limited sample size, which may affect the statistical power of the subgroup analyses. Finally, this study was conducted in ASCVD patients enrolled in the United States, which may limit the generalizability of our findings to other countries.

## 5. Conclusions

In patients with ASCVD, CMI was significantly associated with both all-cause and cardiovascular mortality. Notably, the association between CMI and all-cause mortality was nonlinear. These findings underscore the importance of monitoring and managing CMI in patients with ASCVD in clinical practice and suggest that optimizing CMI levels may help reduce the risk of death and improve the long-term prognosis of patients.

## Figures and Tables

**Table 1:** Baseline characteristics of the study participants.

	Total (*n* = 2157)	Q1 (*n* = 532)	Q2 (*n* = 546)	Q3 (*n* = 543)	Q4 (*n* = 536)	*p*
Age, years	66.4 ± 12.6	66.8 ± 13.5	67.5 ± 12.1	67.1 ± 11.5	64.3 ± 12.9	<0.001
Gender						<0.001
Male	1244 (57.7)	269 (50.6)	304 (55.7)	326 (60.0)	345 (64.4)	
Female	913 (42.3)	263 (49.4)	242 (44.3)	217 (40.0)	191 (35.6)	
Race						<0.001
Mexican American	248 (11.5)	33 (6.2)	55 (10.0)	76 (14.0)	84 (15.7)	
Other Hispanic	151 (7.0)	35 (6.6)	37 (6.8)	40 (7.4)	39 (7.3)	
Non-Hispanic White	1205 (55.9)	263 (49.4)	302 (55.3)	303 (55.8)	337 (62.9)	
Non-Hispanic Black	425 (19.7)	165 (31.0)	120 (22.0)	89 (16.4)	51 (9.5)	
Other Race	128 (5.9)	36 (6.8)	32 (5.9)	35 (6.5)	25 (4.7)	
Educational level						0.002
Below high school	377 (17.5)	80 (15.0)	86 (15.8)	106 (19.5)	105 (19.6)	
High school	909 (42.1)	204 (38.4)	235 (43.0)	221 (40.7)	249 (46.5)	
Above high school	871 (40.4)	248 (46.6)	225 (41.2)	216 (39.8)	182 (34.0)	
Marital status						<0.001
Never married	124 (5.8)	45 (8.5)	24 (4.4)	22 (4.0)	33 (6.2)	
Married/living with partner	1284 (59.5)	285 (53.6)	315 (57.7)	342 (63.0)	342 (63.8)	
Divorced, separated, or widowed	749 (34.7)	202 (38.0)	207 (37.9)	179 (33.0)	161 (30.0)	
Family PIR						0.028
<1.3	886 (41.1)	205 (38.5)	208 (38.1)	217 (40.0)	256 (47.8)	
1.3–3.5	812 (37.6)	207 (38.9)	213 (39.0)	208 (38.3)	184 (34.3)	
>3.5	459 (21.3)	120 (22.6)	125 (22.9)	118 (21.7)	96 (17.9)	
Smoking						<0.001
Never	819 (38.0)	225 (42.3)	234 (42.9)	196 (36.1)	164 (30.6)	
Former	874 (40.5)	192 (36.1)	200 (36.6)	239 (44.0)	243 (45.3)	
Current	464 (21.5)	115 (21.6)	112 (20.5)	108 (19.9)	129 (24.1)	
Alcohol consumption						0.494
<12 drinks/year	1054 (48.9)	261 (49.1)	275 (50.4)	271 (49.9)	247 (46.1)	
≥12 drinks/year	1103 (51.1)	271 (50.9)	271 (49.6)	272 (50.1)	289 (53.9)	
MVPA	844 (39.1)	214 (40.2)	214 (39.2)	206 (37.9)	210 (39.2)	0.898
BMI, kg/m^2^	29.9 ± 6.6	26.7 ± 6.0	29.2 ± 6.0	31.1 ± 6.4	32.4 ± 6.7	<0.001
TC, mmol/L	4.8 ± 1.2	4.7 ± 1.1	4.6 ± 1.1	4.7 ± 1.2	5.1 ± 1.3	<0.001
Hypertension	1532 (71.0)	370 (69.6)	371 (68.0)	394 (72.6)	397 (74.1)	0.152
Diabetes	651 (30.2)	97 (18.2)	165 (30.2)	174 (32.0)	215 (40.1)	<0.001
Antihypertensive medication	1446 (67.0)	342 (64.3)	346 (63.4)	374 (68.9)	384 (71.6)	0.011
Antidiabetic medication	308 (14.3)	45 (8.5)	73 (13.4)	77 (14.2)	113 (21.1)	<0.001
Cardiovascular medication	1485 (68.9)	347 (65.2)	355 (65.0)	388 (71.5)	395 (73.7)	0.002

Data are mean ± standard deviation (SD) or frequencies with percentages. PIR: poverty income ratio; MVPA: moderate-to-vigorous physical activity; BMI: body mass index; TC: total cholesterol.

**Table 2:** Association between CMI and mortality in ASCVD patients.

		Model 1		Model 2		Model 3	
	Death, (%)	HR (95% CI)	*p*	HR (95% CI)	*p*	HR (95% CI)	*p*
All-cause mortality							
CMI	887 (41.1)	0.974 (0.973, 0.975)	<0.001	1.135 (1.133, 1.136)	<0.001	1.113 (1.112, 1.115)	<0.001
Q1	204 (38.4)	1.318 (1.315, 1.322)	<0.001	1.350 (1.346, 1.353)	<0.001	1.426 (1.423, 1.430)	<0.001
Q2	214 (39.2)	Ref.		Ref.		Ref.	
Q3	236 (43.5)	1.183 (1.180, 1.186)	<0.001	1.278 (1.274, 1.281)	<0.001	1.332 (1.328, 1.335)	<0.001
Q4	233 (43.5)	1.109 (1.106, 1.111)	<0.001	1.475 (1.471, 1.479)	<0.001	1.426 (1.422, 1.430)	<0.001
Cardiovascular mortality							
CMI	300 (13.9)	1.056 (1.054, 1.057)	<0.001	1.134 (1.132, 1.135)	<0.001	1.064 (1.062, 1.065)	<0.001
Q1	70 (13.2)	1.658 (1.650, 1.665)	<0.001	1.671 (1.664, 1.679)	<0.001	1.678 (1.670, 1.686)	<0.001
Q2	63 (11.5)	Ref.		Ref.		Ref.	
Q3	79 (14.6)	1.333 (1.327, 1.339)	<0.001	1.386 (1.380, 1.393)	<0.001	1.397 (1.391, 1.404)	<0.001
Q4	88 (16.4)	1.714 (1.706, 1.721)	<0.001	1.983 (1.974, 1.991)	<0.001	1.826 (1.818, 1.835)	<0.001

*p* values, HRs, and 95% CIs from Cox regression. Model 1 unadjusted. Model 2 adjusted for age, gender, and race. Model 3 further adjusted for educational level, marital status, family PIR, smoking status, alcohol consumption, MVPA, BMI, TC, hypertension, diabetes, and medication use. CMI: cardiometabolic index; ASCVD: atherosclerotic cardiovascular disease; HR: hazard ratio; 95% CI: 95% confidence interval; PIR: poverty income ratio; MVPA: moderate-to-vigorous physical activity; BMI: body mass index; TC: total cholesterol.

**Table 3:** Subgroup analyses of the association between CMI and mortality in ASCVD patients.

	All-Cause Mortality				Cardiovascular Mortality			
	Death, (%)	HR (95% CI)	*p*	*p* for Interaction	Death, (%)	HR (95% CI)	*p*	*p* for Interaction
Gender				<0.001				<0.001
Male	560 (45.0)	1.065 (1.063, 1.066)	<0.001		201 (16.2)	1.167 (1.164, 1.169)	<0.001	
Female	327 (35.8)	1.201 (1.199, 1.203)	<0.001		99 (10.8)	0.903 (0.900, 0.905)	<0.001	
Educational level				<0.001				<0.001
Below high school	189 (50.1)	1.330 (1.326, 1.333)	<0.001		57 (15.1)	1.491 (1.483, 1.500)	<0.001	
High school	390 (42.9)	1.107 (1.105, 1.109)	<0.001		119 (13.1)	0.800 (0.798, 0.803)	<0.001	
Above high school	308 (35.4)	1.054 (1.052, 1.056)	<0.001		124 (14.2)	1.219 (1.217, 1.222)	<0.001	
Marital status				<0.001				<0.001
Never married	36 (29.0)	0.363 (0.359, 0.367)	<0.001		16 (12.9)	0.163 (0.156, 1.170)	<0.001	
Married/living with partner	483 (37.6)	1.142 (1.140, 1.143)	<0.001		162 (12.6)	1.057 (1.054, 1.059)	<0.001	
Divorced, separated, or widowed	368 (49.1)	1.030 (1.028, 1.032)	<0.001		122 (16.3)	1.073 (1.069, 1.076)	<0.001	
Family PIR				<0.001				<0.001
<1.3	371 (41.9)	0.995 (0.993, 0.998)	<0.001		120 (13.5)	0.877 (0.874, 0.880)	<0.001	
1.3–3.5	362 (44.6)	1.255 (1.253, 1.257)	<0.001		121 (14.9)	1.230 (1.226, 1.233)	<0.001	
>3.5	154 (33.6)	1.003 (1.000, 1.006)	0.061		59 (12.9)	1.127 (1.122, 1.131)	<0.001	
Smoking status				<0.001				<0.001
Never	304 (37.1)	1.062 (1.060, 1.065)	<0.001		107 (13.1)	0.914 (0.910, 0.918)	<0.001	
Former	408 (46.7)	1.137 (1.135, 1.139)	<0.001		141 (16.1)	1.167 (1.164, 1.171)	<0.001	
Current	175 (37.7)	1.005 (1.002, 1.008)	0.002		52 (11.2)	0.947 (0.943, 0.951)	<0.001	
Alcohol consumption				<0.001				<0.001
<12 drinks/year	408 (38.7)	1.246 (1.244, 1.248)	<0.001		149 (14.1)	1.107 (1.105, 1.110)	<0.001	
≥12 drinks/year	479 (43.4)	0.968 (0.966, 0.970)	<0.001		151 (13.7)	1.005 (1.002, 1.008)	0.001	
MVPA				<0.001				<0.001
No	577 (44.0)	1.091 (1.090, 1.093)	<0.001		205 (15.6)	1.007 (1.004, 1.009)	<0.001	
Yes	310 (36.7)	1.149 (1.147, 1.151)	<0.001		95 (11.3)	1.142 (1.139, 1.146)	<0.001	
Hypertension				<0.001				<0.001
No	249 (39.8)	1.083 (1.081, 1.086)	<0.001		79 (12.6)	1.021 (1.016, 1.027)	<0.001	
Yes	638 (41.6)	1.091 (1.089, 1.092)	<0.001		221 (14.3)	1.071 (1.069, 1.073)	<0.001	
Diabetes				<0.001				<0.001
No	610 (40.5)	1.078 (1.076, 1.080)	<0.001		197 (13.1)	1.030 (1.027, 1.033)	<0.001	
Yes	277 (42.6)	1.129 (1.128, 1.131)	<0.001		103 (15.8)	1.072 (1.069, 1.075)	<0.001	

*p* values, HRs, and 95% CIs from Cox regression adjusted for age, gender, race, educational level, marital status, family PIR, smoking status, alcohol consumption, MVPA, BMI, TC, hypertension, diabetes, and medication use. CMI: cardiometabolic index; ASCVD: atherosclerotic cardiovascular disease; HR: hazard ratio; 95% CI: 95% confidence interval; PIR: poverty income ratio; MVPA: moderate-to-vigorous physical activity; BMI: body mass index; TC: total cholesterol.

## Data Availability

Data used for this study are available on the NHANES website: https://wwwn.cdc.gov/nchs/nhanes/ (accessed on 1 December 2024).

## Abbreviations

ASCVD	atherosclerotic cardiovascular disease
CMI	cardiometabolic index
NHANES	National Health and Nutrition Examination Survey
MetS	metabolic syndrome
WHtR	waist-to-height ratio
TG	triglycerides
HDL-C	high-density lipoprotein cholesterol
NCHS	National Center for Health Statistics
CDC	Centers for Disease Control and Prevention
WC	waist circumference
ICD	International Classification of Diseases
PIR	poverty income ratio
BMI	body mass index
TC	total cholesterol
MVPA	moderate-to-vigorous physical activity
SD	standard deviation
IQR	interquartile range
ANOVA	one-way analysis of variance
HR	hazard ratio
CI	confidence interval
MESA	Multi-Ethnic Study of Atherosclerosis
